# Enhancing Rural Health Dialogue: The Crucial Role of Reflective Practice in Family Physician Involvement

**DOI:** 10.7759/cureus.48380

**Published:** 2023-11-06

**Authors:** Ryuichi Ohta, Chiaki Sano

**Affiliations:** 1 Community Care, Unnan City Hospital, Unnan, JPN; 2 Community Medicine Management, Shimane University Faculty of Medicine, Izumo, JPN

**Keywords:** chronic disease prevention, interprofessional collaboration, medical knowledge dissemination, cultural understanding, healthcare resources, community engagement, rural health dialogues, help-seeking behaviors, rural healthcare, family physicians

## Abstract

Health dialogue plays a pivotal role in sustaining rural communities by enhancing help-seeking behaviors (HSBs). This article delves deep into how family physicians accentuate the efficacy of rural health dialogues, prompting rural citizens to evaluate and adapt their current HSBs critically. Establishing a foundation of trust in rural family physicians significantly influences the motivation for refined HSBs. Additionally, such engagements optimize the application of limited healthcare resources. For these outcomes to be realized, family physicians must amplify their communication and leadership abilities, and confront the inherent challenges of disseminating contemporary medical evidence in rural domains.

## Editorial

Introduction

Family physicians' role in rural communities' fabric should be emphasized. As the demographic composition of these areas leans towards aging, there is a pronounced need for focused medical care, with a particular emphasis on preventing chronic diseases [1]. This demographic shift accentuates the necessity for family physicians to be deeply entrenched in the socio-cultural dynamics of the older rural populace, orchestrating health interventions via community collaboration [2]. This editorial underscores the importance of incorporating reflective practices in rural family medicine, advocating for a synergy between healthcare professionals and the community through health dialogues. 

The role of rural health dialogue

Central to the discourse on rural health is the dialogue - a platform that fosters a symbiotic interaction between healthcare professionals and the community [3]. Spearheaded by family physicians, these dialogues pave the way for rural inhabitants to introspect on their existing help-seeking behaviors (HSBs). With their expansive knowledge and holistic care approach, family physicians shepherd rural residents toward an enhanced comprehension and potential reconfiguration of their HSBs. As evidenced by prior research, a considerable portion of the rural populace grapples with confidence regarding their HSBs, often culminating in suboptimal healthcare decisions [3]. Persistent engagement in these health dialogues can catalyze a more proactive involvement of the rural community in their health decisions.

Family physicians, championing the core tenets of their specialization, should approach every patient encounter as a golden opportunity for health promotion or disease prevention [4]. These dialogues afford them a window into the community's collective psyche regarding health and disease narratives. Armed with these insights, they can tailor interventions that resonate with the community's lived experiences and daily life, fostering more effective health behaviors.

Building trust and enhancing HSBs

The regularity and consistency of health dialogues in rural settings lay the groundwork for an enduring trust between rural citizens and family physicians [3]. While the innate curiosity of rural inhabitants propels them towards acquiring knowledge about health and new HSBs, the perceived chasm from medical professionals can be a deterrent. The continuous rhythm of these dialogues can dispel such inhibitions, nurturing a community better equipped to make informed health decisions [3]. Aligned with the principles of family medicine, physicians are implored to immerse themselves in the unique challenges rural communities face. This close-knit engagement fosters a more genuine understanding, bridging psychological and informational gaps, thus catalyzing more effective HSBs.

Optimizing limited healthcare resources

Rural health dialogues serve as catalysts, motivating citizens to utilize the available healthcare resources judiciously. With their depth of knowledge, family physicians guide these dialogues towards actionable insights, suggesting avenues to harness these resources for preventive and promotive health. Key to this endeavor is the comprehension of the socio-cultural fabric of the community [4]. Such open forums embolden rural citizens to voice their concerns, challenges, and aspirations, allowing for healthcare interventions that are both personalized and impactful.

Overcoming the evidence pipeline challenge

For the rural health dialogue to be impactful, family physicians must foster an environment of trust through adept communication and keep abreast of the latest medical developments [3]. The metaphor of the "evidence pipeline with leakage" underscores the challenge and importance of translating cutting-edge academic research into actionable insights for the community. Family physicians stand at this critical juncture, ensuring that rural communities are kept in the evolving landscape of medical knowledge.

Embodying the community-centric ethos of family medicine, physicians should position themselves as more than just healthcare providers; they are allies, partners, and advocates [5]. Their role transcends disease-focused interventions, emphasizing a holistic, person-centric care paradigm. As leaders, they are poised to drive collaborations, fostering an environment of mutual respect, understanding, and shared goals.

Conclusion

Family physicians stand as pillars in the edifice of rural healthcare, orchestrating meaningful health dialogues that bridge divides, build trust, and enhance community health behaviors. Their continuous and profound engagement in these dialogues ensures that rural communities are not just passive recipients but active stakeholders in their health narratives. Family physicians are carving out a more integrated, responsive, and effective rural healthcare mosaic through their leadership, expertise, and dedication.
